# Fluid-Improved Particle Swarm Optimization for Parameter Optimization of XRD-Based ***Os Draconis*** Identification Model

**DOI:** 10.3390/molecules31162937

**Published:** 2026-08-21

**Authors:** Yuchen Wang, Hongyan Zhai, Jimin Deng, Lu Cheng, Ye Tao, Jinfeng Chen, Min Tang, Kang Wang, Yazhong Zhang

**Affiliations:** 1 School of Software, North China University of Water Resources and Electric Power, Zhengzhou 450046, China; wangyuchen1@ncwu.edu.cn; 2 Anhui Institute for Food and Drug Inspection and Research, Hefei 230051, China; hongyanzhai@hotmail.com (H.Z.); yanghanissen@126.com (J.D.); 18815519639@163.com (L.C.); 13856080281@163.com (Y.T.); chenjinfeng1121@163.com (J.C.); tm3221dl@163.com (M.T.); 3 Zhongke Hefei Institute of Technology Innovation Engineering, Hefei 230088, China

**Keywords:** *Os Draconis*, XRD, parameter optimization, HIIPSO, PSO, fish schooling behaviour, Voronoi topology

## Abstract

During the X-ray Diffraction (XRD) identification of the traditional Chinese medicine *Os Draconis*, the identification model often suffers from limited classification accuracy due to the difficulty in determining optimal parameters. To address this issue, this paper proposes a Hydrodynamic Improved Particle Swarm Optimization (HIIPSO) algorithm for the deep optimization of model parameters. In practical identification scenarios, the high complexity of XRD data poses severe challenges to the convergence speed and global search capability of optimization algorithms. To enhance model performance, this study introduces the interaction mechanism from fluid dynamics into the particle swarm optimization process. Specifically, HIIPSO incorporates a Voronoi neighbor topology to enhance population diversity and spatial distribution rationality. Concurrently, a hydrodynamic interaction mechanism is constructed to simulate the cooperative behavior of particles in a fluid environment, thereby effectively preventing the algorithm from falling into local optima. A theoretical analysis of the computational complexity of the HIIPSO algorithm in the parameter search task for XRD identification models was conducted, confirming that it falls within an ideal range for engineering applications. Statistical analysis of the experimental results demonstrates that, in the parameter optimization task for the *Os Draconis* identification model, the HIIPSO algorithm significantly outperforms traditional and other baseline algorithms across key metrics, including the optimal value, mean, standard deviation, and median of the objective function. The experimental data indicates that the HIIPSO algorithm can substantially improve the robustness and identification accuracy of the XRD-based *Os Draconis* identification model, making it an optimal solution for parameter optimization problems in the digital identification of complex mineral-based traditional Chinese medicines.

## 1. Introduction

As a traditional mineral-based Chinese medicine, the authenticity identification of *Os Draconis* is directly related to the safety and efficacy of clinical medication. In recent years, utilizing X-ray Diffraction (XRD) technology to obtain the crystal structure characteristics of substances, combined with machine learning or deep learning to construct digital identification models, has become a research hotspot in this field. However, XRD data possess complex characteristics, such as high dimensionality, non-linearity, and severe overlapping of diffraction peaks, which pose significant challenges to the selection of hyperparameters in identification models: the search space contains numerous local extrema, making traditional manual tuning or exhaustive search methods often unable to balance global optimization efficiency with the precise matching of model parameters.

Particle Swarm Optimization (PSO), as an evolutionary computation technique simulating swarm intelligence, demonstrates natural applicability in handling such high-dimensional non-linear optimization problems due to its advantages of being gradient-free, having a fast convergence rate, and being easily parallelizable. PSO can achieve efficient searching within complex parameter landscapes through cooperation and competition among particles. Nevertheless, when facing the highly complex objective functions induced by XRD data, the standard PSO algorithm is prone to “premature convergence” phenomena in the later stages of evolution, such as the loss of population diversity and entrapment in local optima. Therefore, exploring improved optimization algorithms that can enhance global search capabilities while maintaining population vitality is of great significance for improving the identification accuracy and robustness of XRD-based *Os Draconis* identification models.

In this context, we identified Particle Swarm Optimization (PSO) [1] as a highly suitable method. Inspired by the foraging behavior of bird flocks, this evolutionary computation technique can derive robust solutions through a stochastic and self-adaptive process. Despite its advantages in low resource consumption and global search capabilities, the standard PSO algorithm suffers from inherent limitations, notably premature convergence and the loss of population diversity. To overcome these issues, numerous PSO variants have been proposed, primarily focusing on four improvement strategies. First, convergence speed can be directly enhanced through modified learning strategies, such as Peer-learning PSO [2] and Orthogonal Learning PSO [3]. Second, parameter tuning—specifically, adjusting the inertia weight and acceleration coefficients—has been widely adopted to influence the convergence rate [4,5,6,7]. Third, hybrid optimization frameworks integrate PSO with other algorithms, including Genetic Algorithms (GA) [8], Extremal Optimization (EO) [9], and Ant Colony Optimization (ACO) [10]. These hybrid models leverage the strong exploration capabilities of PSO alongside the exploitation strengths of the integrated algorithms [11]. Finally, researchers have investigated particle neighbor topologies to boost the exploration capacity of PSO [12]. Notable topologically enhanced variants include the Fully Informed Particle Swarm (FIPS) [13] and Dynamic Multi-Swarm Particle Swarm Optimization (DMSPSO) [14].

Building upon these foundations, this paper investigates the hydrodynamic interactions within fluid environments and proposes a Hydrodynamic Interaction Improved Particle Swarm Optimization (HIIPSO) algorithm for multi-agent learning. Fundamentally, HIIPSO is a neighbor topology-enhanced PSO variant. While previous studies have demonstrated the efficacy of Voronoi diagrams in modeling hydrodynamic interactions, most existing research relies on the conventional three-A rules (avoidance, alignment, and attraction) for neighbor selection [15]. In contrast, our approach explicitly organizes the neighbor topology using Voronoi diagrams. This design is directly inspired by real-world underwater multi-agent systems, specifically the schooling behavior of fish.

The remainder of this paper is structured as follows: Section 2 reviews the foundational concepts of the basic PSO algorithm, fish schooling behavior, and the definition of Voronoi diagrams. Section 3 provides a detailed description of the HIIPSO algorithm, including the components of the newly introduced terms, alongside a computational complexity analysis. Section 4 presents the experimental results, and Section 5 concludes the paper.

## 2. Background and Related Techniques

This section reviews the foundational research pertinent to our proposed methodology, specifically focusing on the fundamental Particle Swarm Optimization (PSO) algorithm, biological studies on fish behavior, and the Voronoi neighbor topology observed in fish schools. These concepts are elaborated in the following subsections.

### 2.1. Particle Swarm Optimization

The PSO algorithm seeks optimal solutions by iteratively updating the positions of particles within a search space. The trajectory of each particle is governed by its velocity. Throughout the evolutionary process, every particle keeps track of its historical best position, denoted as pbest (particle best). Among all the pbest positions within the swarm, the most optimal one is designated as the gbest (global best). In the standard PSO framework, the velocity and position update equations are formulated as follows:

(1) $$v_{i}^{k+1}=\omega v_{i}^{k}+c_{1}\times r_{1}\times \left({\mathrm{pbest}}_{i}^{k}-p_{i}^{k}\right)+c_{2}\times r_{2}\times \left({\mathrm{gbest}}_{i}^{k}-p_{i}^{k}\right)$$

(2) $$p_{i}^{k+1}=p_{i}^{k}+v_{i}^{k+1}$$

Here, k represents the current iteration number, while r1, r2 are random variables uniformly distributed in the range [0, 1]. The parameters c1 and c2 serve as acceleration coefficients that guide the particle toward the personal best location pbestik and global best location ${\mathrm{gbest}}_{i}^{k}$, respectively. The updated velocity is essentially a weighted combination of these two optimal positions. Additionally, the inertia weight ω regulates the influence of the particle’s previous velocity on its current movement. Ultimately, the new position of the target particle is determined by adding the updated velocity to its previous position.

### 2.2. Fish Behavior

A primary distinction between the standard PSO and our proposed improved algorithm lies in the neighbor topology. To effectively solve our tasks, under fluid environmental constraints, drawing inspiration from bionics is highly advantageous. Multi-agent collective behaviors are prevalent in various species, including mosquito swarms [16], starling flocks [17], and fish schools. Due to their exceptional visual acuity and aerial maneuverability, birds typically interact with a larger number of individuals, and their neighbor topologies are generally governed by the well-known three-A rules (alignment, attraction, and avoidance).

**Figure 1:**
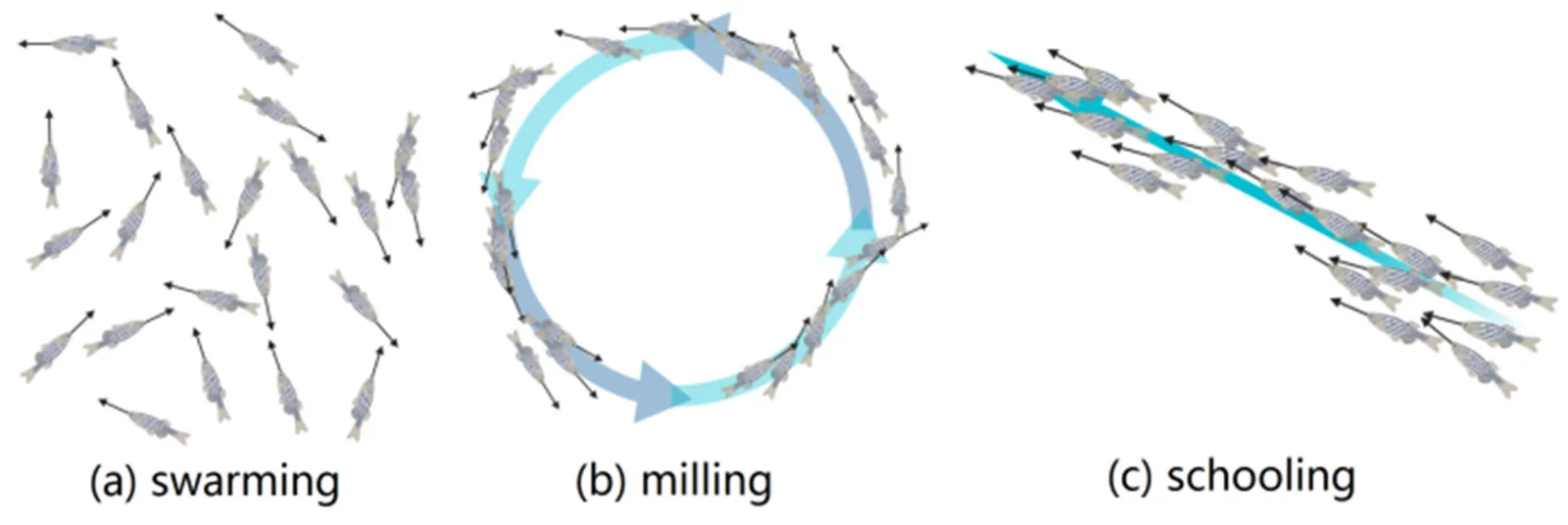
Distinct behavioral patterns observed in fish schools.

Conversely, fish schools operate under fundamentally different organizational mechanisms [18]. Unlike birds, fish perceive the movement of their neighbors primarily through their lateral line system—a sensory organ equipped with hair cells located along their bodies. Although the effective range of hydrodynamic interactions in water is narrower than avian visual perception, it is highly sensitive and perfectly suited for rapid communication with immediate neighbors. Consequently, fish schools exhibit distinct behavioral patterns, such as swarming, milling, and schooling, as illustrated in Figure 1.

Recent advancements in fish school bionomics offer novel insights into organizing neighbor topologies. Under the traditional three-A rules, internal individuals often interact with an excessive number of neighbors, which can cause them to lose their individual characteristics and lead to premature convergence. In contrast, the behavioral rules governing fish schools effectively limit the number of neighbors for internal individuals. This biological constraint prevents information overload and maintains population diversity, thereby providing a valuable mechanism for enhancing the performance of the basic PSO algorithm.

### 2.3. Voronoi Neighbor Topology

Research has demonstrated that fish schools naturally utilize Voronoi neighbors to organize their spatial topology [19,20]. A Voronoi diagram partitions a plane into distinct regions based on the distance to a predefined set of points within that plane. These points, referred to as seeds, agents, or generators, are specified in advance. For each seed, a corresponding region is formed, comprising all spatial points that are closer to that specific seed than to any other. These individual regions are known as Voronoi cells. Mathematically, the Voronoi diagram of a point set is the dual graph of its Delaunay triangulation.

The formal definition of a Voronoi diagram is expressed as follows:

(3) $$R_{k}=\left\{x\in X|d\left(x,P_{k}\right)\leq d\left(x,P_{j}\right)\mathrm{for}\;\mathrm{all}\;j\neq k\right\}$$

Here, $P_{k}$ represents an ordered collection of agents within the space X. The function $d\left(x,P\right)=\left\{d\left(x,p\right)|p\in P\right\}$ denotes the distance between a given point and the subset. Consequently, any point in X whose distance to the current agent $P_{k}$ is less than or equal to its distance to any other agent $P_{j}$ belongs to the Voronoi region $R_{k}$.

In this study, we propose adopting the Voronoi neighbor as a novel neighbor topology. Specifically, if the Voronoi regions of two agents share a boundary (i.e., they are adjacent), these two agents are designated as Voronoi neighbors. Under this topological configuration, agents located in edge Voronoi regions typically possess fewer neighbors. Conversely, internal agents have a greater number of neighbors; however, this quantity remains significantly lower compared to the topology governed by the traditional three-A rules.

## 3. Proposed Algorithm

This section first outlines the problem statement and subsequently details our proposed approach to address it.

Problem Statement:

Consider a set of *n* agents operating in a two-dimensional, obstacle-free environment, aiming to perform single-objective optimization to fulfill various underwater application goals. Given the critical role of underwater hydrodynamics, any applied methodology must be iteratively influenced by hydrodynamic forces. As agents navigate the underwater environment, each agent generates a polarized potential field that decays with distance. Notably, the potential field at the rear of the agent is stronger than that at the front. The primary objective is to develop an algorithm that achieves superior performance and faster convergence while adhering to these hydrodynamic constraints.

To achieve this, a hydrodynamic interaction improvement strategy is introduced. Within the fundamental displacement-velocity framework, hydrodynamic interactions between topological structures are integrated into the particle swarm algorithm. Through the incorporation of these new terms, the algorithm exhibits enhanced adaptability while preserving the intrinsic characteristics of fish schooling. The comprehensive improved optimization model is formulated as follows:

(4) $$\begin{array}{c} v_{i}^{k+1}=\omega v_{i}^{k}+c_{1}\times r_{1}\times \left({\mathrm{pbest}}_{i}^{k}-p_{i}^{k}\right)\;+c_{2}\times r_{2}\times \left({\mathrm{gbest}}_{i}^{k}-p_{i}^{k}\right)+c_{3}\times r_{3}\times H_{i}^{k} \end{array}$$

(5) $$p_{i}^{k+1}=p_{i}^{k}+v_{i}^{k+1}$$

The newly introduced hydrodynamic interaction term is modulated by a random factor $r_{3}$ and a hydrodynamic interaction acceleration learning factor $c_{3}$. Within this topologically enhanced optimization framework, a novel component—the hydrodynamic interaction velocity vector—is defined in the polynomial as follows:

(6) $$H_{i}^{k}=e_{i}^{\theta }\times v_{i}^{h}$$

The hydrodynamic interaction velocity vector is calculated as the product of the orientation vector $e_{i}^{\theta }$ and the average velocity of the Voronoi neighbors, $v_{i}^{h}$. These variables are defined as follows:

(7) $$e_{i}^{\theta }=\left(A+R\right)\times e$$

(8) $$v_{i}^{h}=\frac{1}{N}\sum_{j\in R_{i}}v_{j}$$

In these equations, $R_{i}$ denotes the set of Voronoi neighbors for the focal particle, and N represents the total number of these neighbors. The orientation of the focal particle is influenced by two components: the alignment term A and the rotational hydrodynamic interaction term R. A detailed explanation of these terms is provided in the subsequent subsections.

### 3.1. Alignment Term

As a representative swarm intelligence algorithm, HIIPSO incorporates an alignment mechanism to regulate particle dynamics. This term adjusts the state of each particle based on information from its Voronoi neighbors, thereby promoting the overall coherence of the swarm. The mathematical formulation of the alignment term is presented in Equation (9):

(9) $$A=\sum_{j\in R_{i}}\left(1+\cos\alpha \right)\left(d_{ij}\sin\alpha +I_{1}\sin\gamma \right)$$

**Figure 2:**
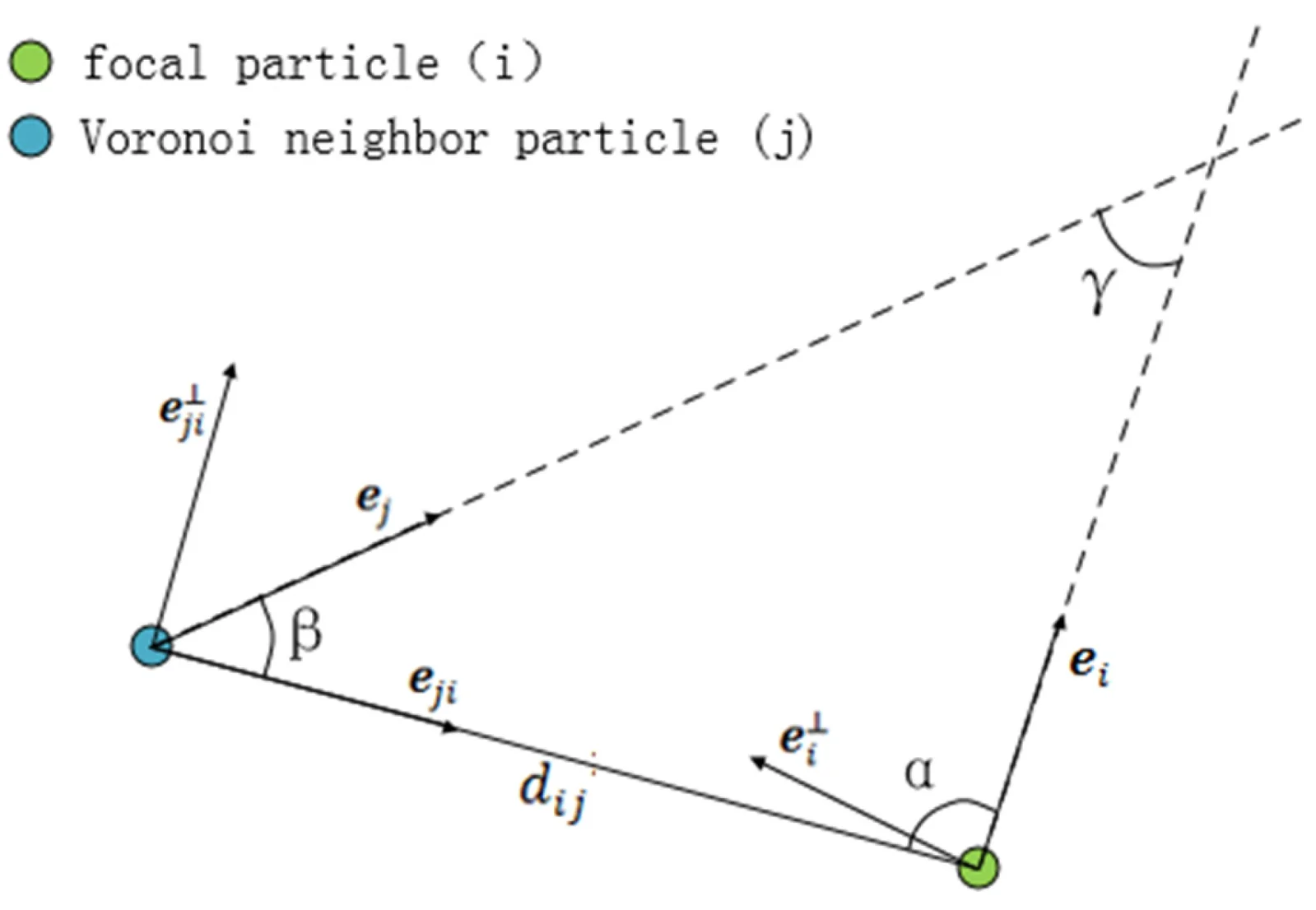
Hydrodynamic interactions between the focal particle and its Voronoi neighbor particles.

In this equation, $I_{1}$ represents the alignment intensity parameter, and $d_{ij}$ denotes the Euclidean distance between the focal particle and its Voronoi neighbor. The geometric angles α, β, and γ are illustrated in Figure 2. To account for hydrodynamic interaction characteristics, a directional weight of $\left(1+\cos\alpha \right)$ is embedded within the alignment term. Physically, this reflects the fact that each swimming fish generates a dipolar flow field, making the hydrodynamic influence in the wake (the rear of the particle) significantly more pronounced.

### 3.2. Rotational Hydrodynamic Interaction Term

The rotational hydrodynamic interaction term, denoted as R, quantifies the effect of the surrounding water flow on the orientation of the agents. It is expressed as:

(10) $$R=\sum_{j\in R_{i}}e_{i}\times \nabla u_{ji}\times e_{i}^{⊥}$$

Here, $u_{ji}$ represents the velocity field induced by the j-th Voronoi neighbor at the position of the focal particle. This induced velocity is calculated using Equation (11):

(11) $$u_{ji}=\frac{I_{3}}{\pi }\times \frac{e_{ji}\sin\beta +e_{ji}^{⊥}\cos\beta }{{d_{ij}}^{2}}$$

where $I_{3}$ is the dipole intensity parameter, while $e_{ji}$ and $e_{ji}^{⊥}$ correspond to the polar coordinate components defined within the local reference frame of the neighboring particle.

### 3.3. Adaptive Adjustment of Inertial Weight and Learning Factor

The inertial weight factor ω is designed to fluctuate dynamically in response to the spatial compactness of the swarm. To prevent particles from clustering too closely—thereby simulating the avoidance of physical collisions among fish—a higher inertial weight is required. The self-adaptive calculation of ω is formulated as follows:

(12) $$\omega =\omega_{max}-\frac{k}{{iter}_{max}}\left(\omega_{max}-\omega_{min}\right)+r\times e^{-E}$$

In this expression, $\omega_{max}$ and $\omega_{min}$ define the dynamic bounds of the linearly decreasing weight, representing its initial and final values, respectively. The variable r is a random seed drawn from the interval $\left(0,1\right)$, while k and ${iter}_{max}$ denote the current and maximum iteration numbers, respectively. $e^{-E}$ denotes the normalized kinetic energy of the particle swarm. By incorporating the kinetic energy of the swarm, the inertia weight decays with a built-in buffer, allowing it to adaptively respond to the swarm’s kinetic state. This self-adaptive updating mechanism is highly beneficial for enhancing both the precision and the convergence rate of the algorithm.

Furthermore, the learning factor $c_{3}$, associated with the hydrodynamic interaction enhancement term, plays a crucial role in preserving swarm diversity. Its dynamic update strategy is defined by Equation (13):

(13) $$\left\{\begin{array}{c} c_{3}=\left(1-\omega \right)\;\;\;\;\;\;\;\left(k\leq \frac{2}{3}{\mathrm{iter}}_{max}\right)\;\\ c_{3}=0\;\;\;\;\;\;\;\;\;\;\;\;\;\;\;\;\;\;\;\;\left(k>\frac{2}{3}{\mathrm{iter}}_{max}\right)\; \end{array}\right.$$

During the iterative process, the gradual reduction in ω naturally leads to a decline in particle diversity. To counteract this, $c_{3}$ is amplified during the early stages of the search to help the algorithm escape local optima. In the later stages, the priority shifts toward refining the solution’s accuracy. It is empirically assumed that after completing two-thirds of the maximum iterations, the algorithm has a high probability of converging toward the global optimum. Consequently, $c_{3}$ is deactivated (set to zero) in this final phase, as maintaining population diversity is no longer necessary.

### 3.4. Computational Complexity Analysis

**Figure 3:**
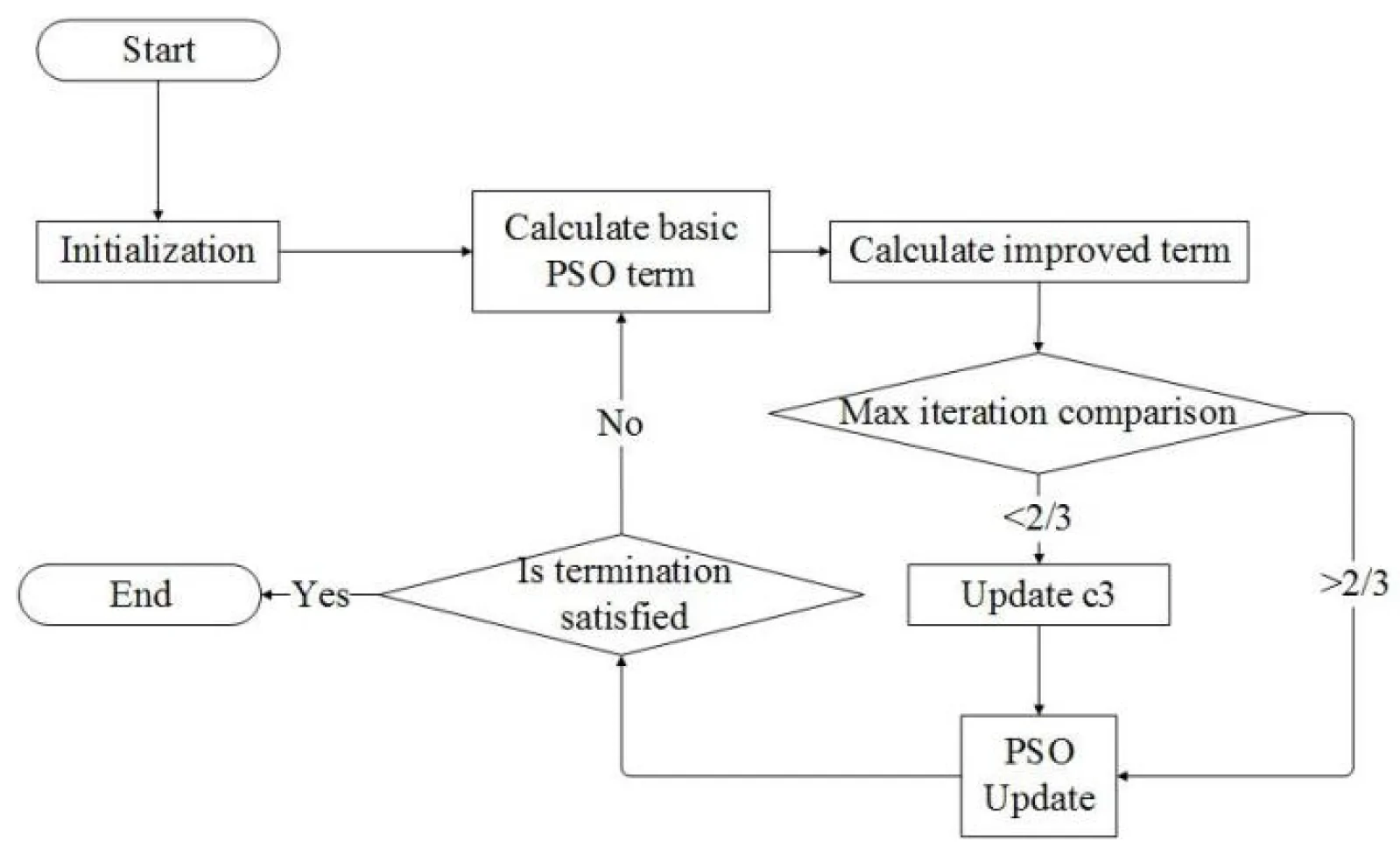
Flowchart of the proposed HIIPSO algorithm.

Evaluating the computational complexity of the proposed algorithm is both essential and necessary. To facilitate this analysis, the flowchart of the HIIPSO algorithm is illustrated in Figure 3. The detailed execution procedure of HIIPSO can be outlined through the following sequential steps:

Step 1: Initialize the algorithm parameters and the initial positions of the particle swarm.

Step 2: Construct the Voronoi neighbor topology for the current particle swarm.

Step 3: Identify the personal best position ${\mathrm{pbest}}_{i}^{k}$ and the global best position ${\mathrm{gbest}}_{i}^{k}$ for each particle.

Step 4: Compute the alignment term A and the rotational hydrodynamic interaction term R based on the Voronoi neighbor.

Step 5: Calculate the orientation vector $e_{i}^{\theta }$ and the average velocity of the Voronoi neighbors $v_{i}^{h}$.

Step 6: Determine the hydrodynamic interaction velocity $H_{i}^{k}$.

Step 7: Update the inertial weight factor ω.

Step 8: If the current iteration count does not exceed two-thirds of the maximum iterations, update the hydrodynamic interaction learning factor.

Step 9: Update the velocity and position of the particles. Evaluate the termination criteria; if unmet, return to Step 2; otherwise, output the final optimization results.

Let n denote the number of particles and k represent the total number of iterations. The computational cost of each step is analyzed as follows:

Step 1 requires one operation for n times initializing particle positions, alongside constant-time operations for parameter initialization. Step 2 demands one operation for n times to construct the Voronoi topology for the current iteration. Step 3 involves O(nlogn) operations to determine the historical best locations and O(nlogn) operations to identify the global best location. Step 4 requires O(n) operations to compute both A and R. Step 5 entails constant-time operations for the orientation vector and O(n) operations for calculating the average neighbor velocity. Step 6 and Step 7 each require only constant-time operations. Step 8 involves constant-time logical judgments and updates. Finally, Step 9 requires O(n) operations to update the velocity and position of the entire swarm.

Considering that the termination condition is evaluated k times throughout the evolutionary process, the overall computational complexity of the proposed HIIPSO algorithm is O(knlogn). This complexity remains at the same level as that of the standard PSO algorithm, indicating its feasibility for practical engineering applications.

## 4. Experimental Setup for XRD-Based ***Os Draconis*** Identification Parameter Optimization

**Figure 4:**
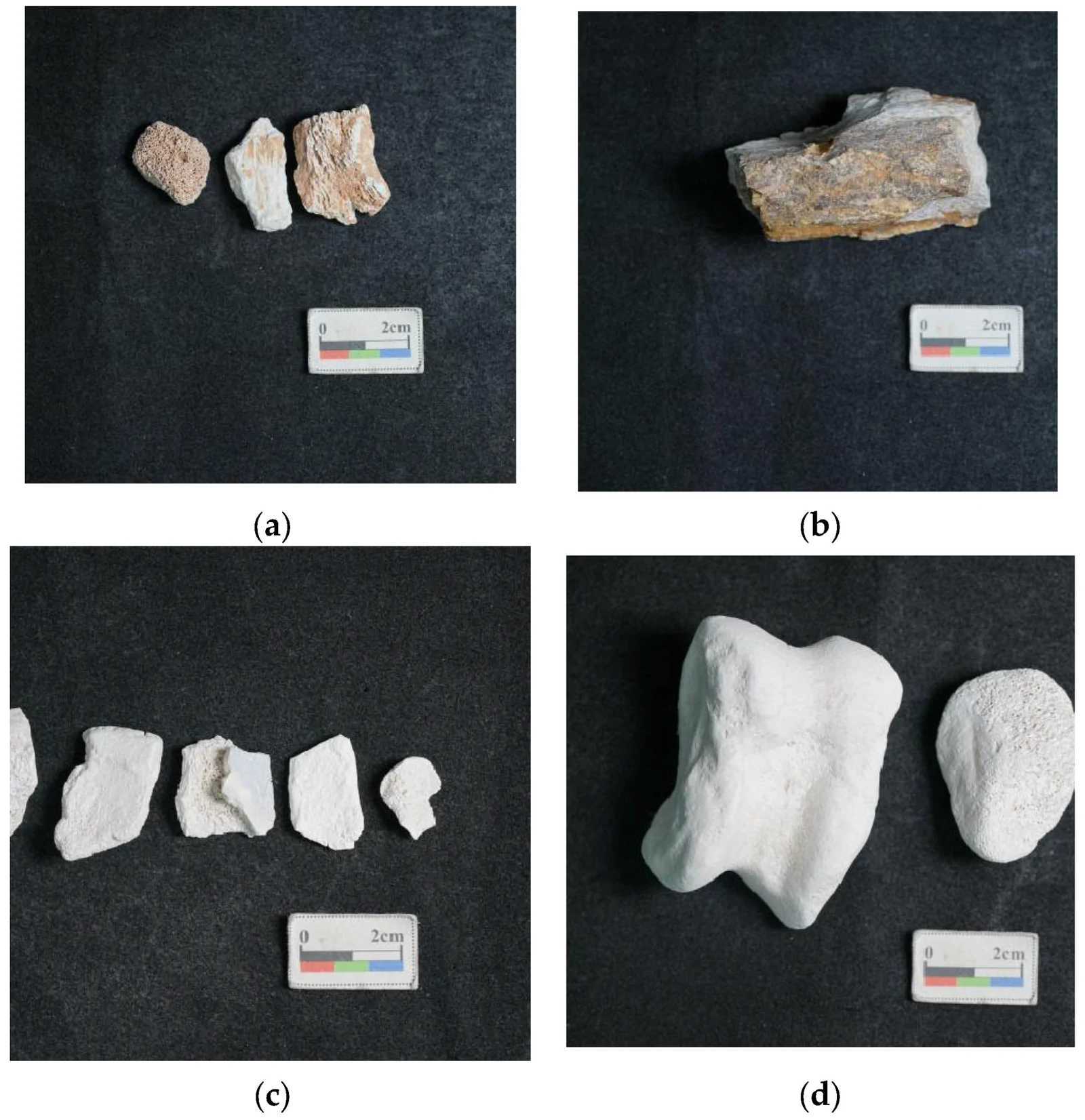
Test samples used in the experiment. Panels (**a**,**b**) display authentic *Os Draconis*, while panels (**c**,**d**) present counterfeit samples.

To validate the effectiveness of the proposed method, comparative experiments were conducted against several state-of-the-art and highly relevant semi-heuristic algorithms. These include Differential Evolution (DE) [21], Artificial Bee Colony (ABC) [22], Genetic Algorithm (GA) [8], and Firefly Swarm Optimization (FSO) [23]. All experiments utilized authentic *Os Draconis* samples as the primary data source, as illustrated in Figure 4.

All computational experiments were executed on a 64-bit Windows 7 operating system, equipped with an Intel(R) Core(TM) i7-3770 processor (3.4 GHz) and 8 GB of RAM. The algorithms were implemented using MATLAB R2017b. For data acquisition, a state-of-the-art intelligent X-ray Diffractometer (XRD) (Model SmartLab 3, Rigaku Corporation, Akishima, Japan) was employed.

To construct the objective functions, the diffraction angle range of the *Os Draconis* samples was divided into five equal intervals. The experimental XRD data corresponding to each interval were assigned as coefficients for distinct objective functions, resulting in a total of five different objective functions.

### 4.1. Parameter Settings

To ensure a fair and rigorous comparison, all evaluated algorithms were configured with identical experimental parameters. The maximum number of iterations ${\mathrm{iter}}_{max}$ was uniformly set to 100. The population size was defined as 50 times the number of decision variables. Given that the problem dimension was set to 5, the population size for each algorithm was configured to 250 (5 × 50). Furthermore, each algorithm was independently executed 50 times to guarantee statistical reliability and mitigate the impact of random initialization (Table 1).

The specific parameter values required for the classical algorithms are summarized in Table 2. To ensure a fair comparison, the classical learning algorithms, PSO variants, and the proposed HIIPSO are all executed under the identical hydrodynamic constraints detailed in Section 3.

### 4.2. Experimental Results

To evaluate the performance, the HIIPSO algorithm was independently executed 50 times for each objective function. The statistical outcomes, including the Minimum (Min), Maximum (Max), Standard Deviation (Std), Mean, and Median (Mdn) values, were recorded. Since the theoretically optimal value for each objective function is zero, the output generated by the learning algorithms is evaluated based on its proximity to this ideal value. The statistical metrics are defined as follows:

Min (Best fitness): The lowest fitness value achieved across the 50 independent runs.

Max (Worst fitness): The highest fitness value recorded across the 50 independent runs.

Std (Standard deviation): A metric indicating the dispersion and stability of the obtained solutions.

Mean (Average fitness): The average fitness value across all 50 runs, reflecting the overall precision and solution quality of the algorithm within the given iterations.

Mdn (Median fitness): The median value of the fitness outcomes across the 50 runs.

The experiments were conducted using uniformly scaled problems, and the comparative statistical data between HIIPSO and the classical learning algorithms are presented in Table 3, with the best-performing values highlighted in bold. The experimental results demonstrate that HIIPSO consistently outperforms all the traditional algorithms across the five objective functions.

Furthermore, the proposed algorithm was compared against several state-of-the-art PSO variants, including Enhanced Leader PSO (ELPSO) [24] Chaotic Inertia Weight PSO (CAIWPSO) [25], Chaotic Random Inertia Weight PSO (CRIWPSO) [25], and Time-Varying Acceleration Coefficient PSO (TVAPSO) [26]. For these comparative tests, the maximum number of iterations, problem dimension, and population size were kept constant at 100, 5, and 250, respectively. The statistical comparison between HIIPSO and these PSO variants is detailed in Table 3. The results indicate that HIIPSO similarly achieves superior performance compared to all the enhanced PSO algorithms across the five objective functions.

### 4.3. Authentic-vs-Counterfeit Identification Pipeline and Performance Evaluation

To bridge the HIIPSO algorithm with the practical authentication of *Os Draconis*, a machine-learning-based classification pipeline was established. The detailed identification workflow and sample authentication procedures are outlined below:

1. Ground-Truth Authentication and Sample Preparation. A total of 196 *Os Draconis* raw material samples were collected. The ground-truth identities of all samples were rigorously certified by senior experts in Chinese materia medica in accordance with the standard macroscopic, microscopic, and chemical criteria stipulated in the Pharmacopoeia of the People’s Republic of China.
2. Identification Classifier Mapping. After extracting the XRD spectral features and optimizing the parameters across the five diffraction angle intervals using the proposed HIIPSO algorithm, the optimized parameters were mapped into a classifier. Specifically, the sample dataset was split into a training set and an independent test set using 5-fold cross-validation to construct the authentic-vs-counterfeit decision boundary.
3. Performance Evaluation Metrics. To comprehensively evaluate the practical classification efficacy of the HIIPSO-optimized model, we recorded the confusion matrix and calculated five standard binary classification performance metrics: Accuracy (ACC), Sensitivity (SEN, true positive rate), Specificity (SPE, true negative rate), and the Receiver Operating Characteristic (ROC) curve along with the Area Under the Curve (AUC).

## 5. Conclusions

In this study, we propose a novel optimization algorithm, named the Hydrodynamic Interaction Improved Particle Swarm Optimization (HIIPSO), and apply it to the parameter optimization of the XRD-based *Os Draconis* identification model. Drawing inspiration from natural hydrodynamic interaction characteristics, this algorithm seamlessly integrates these physical mechanisms into the evolutionary framework of the standard Particle Swarm Optimization (PSO). The core innovations of HIIPSO are primarily reflected in two aspects. First, the introduction of the Voronoi neighbor topology presents new possibilities for organizing the spatial distribution of the particle swarm, thereby enhancing the algorithm’s coverage capability in complex solution spaces. Second, by incorporating hydrodynamic interaction analysis within a fluid environment, the velocity update equation of the particles is significantly refined.

A rigorous computational complexity analysis was conducted on the HIIPSO algorithm, confirming that its computational cost remains well within acceptable limits for practical engineering applications. Under identical experimental environments and constraints, HIIPSO was evaluated against both traditional algorithms and several state-of-the-art PSO variants. Statistical evaluations demonstrate that the proposed algorithm significantly outperforms the other comparative learning algorithms across multiple metrics in the vast majority of test scenarios.

Attributed to the enhanced topology and the introduction of the hydrodynamic interaction mechanism, HIIPSO effectively overcomes the premature convergence issue inherent in traditional PSO. When tackling the complex nonlinear parameter search tasks within the XRD Os Draconis identification model, it achieves notably superior optimization depth compared to the baseline algorithms. Overall, the HIIPSO algorithm can substantially elevate the performance of digital identification models for mineral-based traditional Chinese medicines (TCMs), demonstrating broad application prospects and practical value in the automated identification and complex decision-making domains of TCMs. Future research will focus on extending this parameter optimization approach to the XRD identification models of other medicinal materials.

## Figures and Tables

**Table 1:** Parameters settings.

Algorithm	Parameter	Value
HIIPSO	Inertia weight range	(0.4,0.9)
	Learning factor	2
	Alignment intensity	9
	Noise intensity	0.5
	Dipole intensity	0.01
DE	Crossover probability	0.9
	Scaling factor	0.5
ABC	Sole control	50
GA	Crossover rate	0.8
FSO	Attraction coefficient	0.6
	Randomness factor	0.5
	Attraction exponent	1

**Table 2:** Statistical data of HIIPSO and classical learning algorithms.

		HIIPSO	DE	ABC	GA	FSO
$F_{1}$	Min	**1.34 × 10^−20^**	6.79 × 10^−9^	1.66 × 10^−9^	0.00511	7.80 × 10^−6^
	Max	**1.52 × 10^−13^**	2.41 × 10^−7^	4.41 × 10^−8^	0.06721	0.00006
	Std	**1.86 × 10^−14^**	3.62 × 10^−8^	7.66 × 10^−9^	0.01307	1.32 × 10^−5^
	Mean	**5.09 × 10^−15^**	5.85 × 10^−8^	1.24 × 10^−8^	0.01503	0.00002
	Mdn	**2.58 × 10^−16^**	5.62 × 10^−8^	1.04 × 10^−8^	0.00547	0.00002
$F_{2}$	Min	**5.59 × 10^−14^**	5.21 × 10^−4^	0.00378	0.82214	0.00031
	Max	**0.79373**	0.02170	0.15522	1.65 × 10^1^	0.00146
	Std	**0.14098**	0.00488	0.01938	1.68708	0.00027
	Mean	**0.02867**	0.00869	0.03158	1.42754	0.00085
	Mdn	**3.79 × 10^−12^**	0.00844	0.02256	0.86829	0.00100
$F_{3}$	Min	**3.57 × 10^−15^**	9.58 × 10^−6^	0.00050	0.00398	0.00002
	Max	**2.10 × 10^−11^**	0.00012	0.01290	2.90229	0.00020
	Std	**2.72 × 10^−12^**	0.00003	0.00178	0.36322	0.00004
	Mean	**1.19 × 10^−12^**	0.00006	0.00332	0.06989	0.00008
	Mdn	**4.54 × 10^−13^**	0.00004	0.00261	0.02666	0.00007
$F_{4}$	Min	**1.58 × 10^−12^**	1.02564	2.59210	1.04 × 10^4^	3.08 × 10^3^
	Max	**7.25 × 10^−5^**	3.24 × 10^1^	2.88 × 10^1^	9.13 × 10^4^	4.67 × 10^4^
	Std	**9.70 × 10^−6^**	4.99200	6.60152	1.88 × 10^4^	1.08 × 10^4^
	Mean	**2.46 × 10^−6^**	7.65453	1.19 × 10^1^	1.81 × 10^4^	2.01 × 10^4^
	Mdn	**1.08 × 10^−7^**	6.04550	9.31177	1.52 × 10^4^	1.98 × 10^4^
$F_{5}$	Min	**3.08 × 10^−16^**	0.00011	0.00033	8.06 × 10^1^	2.83 × 10^1^
	Max	**1.83 × 10^−7^**	0.00631	0.00690	4.83 × 10^4^	1.61 × 10^3^
	Std	**2.03 × 10^−8^**	0.00141	0.00132	6.34 × 10^3^	3.38 × 10^2^
	Mean	**6.50 × 10^−9^**	0.00089	0.00268	1.99 × 10^3^	4.21 × 10^2^
	Mdn	**5.11 × 10^−10^**	0.00063	0.00308	1.10 × 10^2^	4.82 × 10^2^

**Table 3:** Statistical data of HIIPSO and PSO variants.

		HIIPSO	ELPSO	CAIWPSO	CRIWPSO	TVAPSO
$F_{1}$	Min	**1.34 × 10^−20^**	6.58 × 10^−9^	1.50 × 10^−9^	0.00441	8.21 × 10^−6^
	Max	**1.52 × 10^−13^**	1.93 × 10^−7^	4.24 × 10^−8^	0.06942	0.00006
	Std	**1.86 × 10^−14^**	3.72 × 10^−8^	8.53 × 10^−9^	0.01385	1.16 × 10^−5^
	Mean	**5.09 × 10^−15^**	6.97 × 10^−8^	1.17 × 10^−8^	0.01337	0.00002
	Mdn	**2.58 × 10^−16^**	5.99 × 10^−8^	1.30 × 10^−8^	0.00729	0.00003
$F_{2}$	Min	**5.59 × 10^−14^**	4.94 × 10^−4^	0.00457	0.84638	0.00037
	Max	**0.79373**	0.02085	0.17300	1.42E+01	0.00140
	Std	**0.14098**	0.00424	0.02091	1.74828	0.00035
	Mean	**0.02867**	0.00809	0.02750	1.54536	0.00108
	Mdn	**3.79 × 10^−12^**	0.00924	0.02617	1.15875	0.00117
$F_{3}$	Min	**3.57 × 10^−15^**	7.24 × 10^−6^	0.00056	0.00308	0.00002
	Max	**2.10 × 10^−11^**	0.00015	0.01049	2.33892	0.00020
	Std	**2.72 × 10^−12^**	0.00004	0.00245	0.42413	0.00004
	Mean	**1.19 × 10^−12^**	0.00006	0.00297	0.08644	0.00008
	Mdn	**4.54 × 10^−13^**	0.00004	0.00269	0.02603	0.00008
$F_{4}$	Min	**1.58 × 10^−12^**	1.24764	2.38740	1.42 × 10^5^	3.24 × 10^3^
	Max	**7.25 × 10^−5^**	2.62 × 10^1^	3.69 × 10^1^	1.32 × 10^4^	5.40 × 10^4^
	Std	**9.70 × 10^−6^**	5.78400	6.12896	1.64 × 10^4^	9.27 × 10^3^
	Mean	**2.46 × 10^−6^**	7.57302	1.11 × 10^1^	1.17 × 10^4^	2.00 × 10^4^
	Mdn	**1.08 × 10^−7^**	5.02285	9.43259	1.31 × 10^4^	1.80 × 10^4^
$F_{5}$	Min	**3.08 × 10^−16^**	0.00011	0.00036	7.60 × 10^1^	2.13 × 10^1^
	Max	**1.83 × 10^−7^**	0.00842	0.00533	6.09 × 10^4^	1.60 × 10^3^
	Std	**2.03 × 10^−8^**	0.00102	0.00136	8.60 × 10^3^	3.82 × 10^2^
	Mean	**6.50 × 10^−9^**	0.00100	0.00284	2.12 × 10^3^	4.40 × 10^2^
	Mdn	**5.11 × 10^−10^**	0.00062	0.00293	8.45 × 10^1^	3.92 × 10^2^

## Data Availability

The XRD datasets generated and analyzed during the current study are available from the corresponding author upon reasonable request.

## Abbreviations

The following abbreviations are used in this manuscript:

XRD	X-ray diffraction
HIIPSO	Hydrodynamic Interaction Improved Particle Swarm Optimization
PSO	Particle Swarm Optimization
GA	Genetic Algorithms
EO	Extremal Optimization
ACO	Ant Colony Optimization
FIPS	Fully Informed Particle Swarm
DMSPSO	Dynamic Multi-Swarm Particle Swarm Optimization
